# Radiographic response to neoadjuvant therapy in pleural mesothelioma should serve as a guide for patient selection for cytoreductive operations

**DOI:** 10.3389/fonc.2023.1216999

**Published:** 2023-08-11

**Authors:** Nathaniel Deboever, Nicolas Zhou, Daniel J. McGrail, Katarzyna Tomczak, Jacqueline L. Oliva, Hope A. Feldman, Edwin Parra, Jianjun Zhang, Percy P. Lee, Mara B. Antonoff, Wayne L. Hofstetter, Reza J. Mehran, Ravi Rajaram, David C. Rice, Jack A. Roth, Stephen S. Swisher, Ara A. Vaporciyan, Mehmet Altan, Annikka Weissferdt, Anne S. Tsao, Cara L. Haymaker, Boris Sepesi

**Affiliations:** ^1^Department of Thoracic and Cardiovascular Surgery, University of Texas MD Anderson Cancer Center, Houston, TX, United States; ^2^Department of Bioinformatics and Computational Biology, University of Texas MD Anderson Cancer Center, Houston, TX, United States; ^3^Department of Translational Molecular Pathology, University of Texas MD Anderson Cancer Center, Houston, TX, United States; ^4^Department of Thoracic/Head and Neck Medical Oncology, University of Texas MD Anderson Cancer Center, Houston, TX, United States; ^5^Department of Radiation Oncology, University of Texas MD Anderson Cancer Center, Houston, TX, United States; ^6^Department of Pathology, University of Texas MD Anderson Cancer Center, Houston, TX, United States

**Keywords:** malignant pleural mesothelioma, neoadjuvant therapy, radiographic response, cytoreductive resection, patient-centered care

## Abstract

**Background:**

Malignant pleural mesothelioma (MPM) is associated with poor prognosis despite advances in multimodal therapeutic strategies. While patients with resectable disease may benefit from added survival with oncologic resection, patient selection for mesothelioma operations often relies on both objective and subjective evaluation metrics. We sought to evaluate factors associated with improved overall survival (OS) in patients with mesothelioma who underwent macroscopic complete resection (MCR).

**Methods:**

Patients with MPM who received neoadjuvant therapy and underwent MCR were identified in a prospectively maintained departmental database. Clinicopathologic, blood-based, and radiographic variables were collected and included in a Cox regression analysis (CRA). Response to neoadjuvant therapy was characterized by a change in tumor thickness from pretherapy to preoperative scans using the modified RECIST criteria.

**Results:**

In this study, 99 patients met the inclusion criteria. The median age of the included patients was 64.7 years, who were predominantly men, had smoking and asbestos exposure, and who received neoadjuvant therapy. The median change in tumor thickness following neoadjuvant therapy was –16.5% (interquartile range of -49.7% to +14.2%). CRA demonstrated reduced OS associated with non-epithelioid histology [hazard ratio (HR): 3.06, 95% confidence interval (CI): 1.62–5.78, p < 0.001] and a response to neoadjuvant therapy inferior to the median (HR: 2.70, CI: 1.55–4.72, p < 0.001). Patients who responded poorly (below median) to neoadjuvant therapy had lower median survival (15.8 months compared to 38.2 months, p < 0.001).

**Conclusion:**

Poor response to neoadjuvant therapy in patients with MPM is associated with poor outcomes even following maximum surgical cytoreduction and should warrant a patient-centered discussion regarding goals of care and may therefore help guide further therapeutic decisions.

## 1 Introduction

The prognosis associated with the diagnosis of malignant pleural mesothelioma (MPM) surrounds patient and clinicopathologic factors; however, it remains poor despite robust efforts to modulate oncologic outcomes with novel neoadjuvant therapies (1, 2). With the increased use of multimodal therapy (3), there remains an equipoise in selecting patients who may benefit from resection. While the inception of enhanced recovery pathways reduced surgical morbidity, the convalescence associated with macroscopic complete resection (MCR) is protracted and often associated with decreased quality of life (4) and potential detrimental effects for the receipt of other established or experimental therapies, regardless of whether pleurectomy and decortication or extrapleural pneumonectomy was performed.

The current clinical staging metrics have been insufficient in providing clinicians with objective tools to inform patient-centered prognosis discussions (5). Due to a deficiency of meaningful prognostic measures, multiple groups have suggested scoring systems (6–8) or factors (9) that can help prognostication of this patient population. While these may inform prognosis in patients with MPM often prior to receipt of any therapy, patient and disease characteristics that might affect overall survival in patients undergoing resection for MPM remain underutilized.

Surgical decisions are currently based on patient and disease factors, patient’s wishes, and surgeon’s experience, using insight on the aggressivity and biologic behavior of this disease. Thus, we sought to augment the surgical decision-making process by investigating a series of environmental, clinicopathologic, treatment response, blood-based, and radiographic factors that might reflect disease and host physiology. We aimed to integrate blood-based and radiographic changes that mirror the dynamic pathological milieu impacted by neoadjuvant therapy into our analyses to enhance the surgical decision process for resectable MPM.

## 2 Materials and methods

### 2.1 Study design, population, and treatment

A prospectively maintained single-institution departmental database was retrospectively queried for patients with MPM, evaluated by a multidisciplinary team, who underwent MCR following neoadjuvant therapy between April 2005 and September 2019 at the University of Texas MD Anderson Cancer Center (MDACC). This study was undertaken following approval by MDACC’s Institutional Review Board with a waiver of individual informed consent (PA 2019-1197).

Patient and disease-specific variables obtained included smoking status, asbestos exposure, baseline pulmonary function, and performance status, as well as clinical and pathological stage (adjusted to the eighth edition American Joint Commission on Cancer staging) and histopathology. A subgroup of patients with epithelioid histology was defined *a priori*. Peripheral blood samples were collected prior to the start of neoadjuvant systemic therapy and again following the completion of therapy but prior to surgery. These samples were the source of peripheral blood-based data that included absolute lymphocyte, absolute neutrophil, and platelet counts. These were then used to calculate neutrophil-to-lymphocyte ratio (NLR) and platelet-to-lymphocyte ratio (PLR). Radiographic data were obtained from computed tomography (CT) scans completed prior to the initiation of neoadjuvant systemic therapy, during neoadjuvant systemic therapy, and included the last scan prior to the resection. Tumor thickness was measured according to the revised modified RECIST (mRECIST) (10, 11) criteria. All scans included met the minimum quality standard as suggested by the criteria. The radiographic response to neoadjuvant systemic therapy was categorized by the change in tumor thickness from prior to systemic therapy to the most recent preoperative scan as a percentage change. Tumor response was also stratified using the mRECIST criteria, which defined partial response (PR) as a decrease in tumor thickness of at least 30%, stable disease (SD) as a decrease of <30% or an increase of <20% in tumor thickness, and progressive disease (PD) as an increase in tumor thickness of at least 20%. Overall survival was defined from the initiation of neoadjuvant systemic therapy until date of death or censored at the last follow-up. Stratification of the cohort by 20 months’ survival was performed, which characterized a clinically meaningful threshold, considering the historical median overall survival of patients with resectable MPM, in addition to surgical convalescence (4), and as a comparison to 18 months’ survival achieved in the immunotherapy cohort of Checkmate 743 trial.

### 2.2 Statistical methods

A paired T-test was used to compare the clinical to pathological tumor and nodal statuses in order to assess concordance. A Cox proportional hazards regression model and multivariable linear regression model were used to assess and identify variables that may inform overall survival. Covariates were included in the models if they satisfied Wald’s backward elimination using a probability value threshold of 0.200. The Kaplan–Meier method and a log-rank test were used to further determine the survival probability of patients with MPM stratified by radiographic response to neoadjuvant systemic therapy. A subgroup analysis defined *a priori* was performed and included only patients with epithelioid disease. Collinearity was assessed using variance inflation factors. Analyses were performed using R Studio (version 1.4.1717, PBC, Boston, MA, USA) and GraphPad Prism (version 9.3.1, GraphPad Software, San Diego, CA, USA).

## 3 Results

### 3.1 Environmental, clinicopathologic, circulomic, and radiomic characteristics

There were 99 patients who met the inclusion criteria during the study period. The median age of the patients in this cohort was 64.7 years [interquartile range (IQR): 59.3–68.1]; the majority of the patients were men (n = 74, 74.7%), had smoking exposure (ever smoker, n = 53, 53.5%), and had asbestos exposure (n = 74, 74.7%). Patients were most commonly diagnosed with clinical stage I disease (n = 52, 52.5%) and clinical stage III disease (n = 27, 27.3%). However, upstaging following resection was significantly common (p < 0.001) in 58 patients (58.6%), leading to pathological stage III (n = 55, 55.6%) and IV (n = 23, 23.2%) predominance. This was secondary to a significant rate of upstaging in both tumor (p < 0.001) and nodal statuses (p < 0.001). The cohort included patients who predominantly received a platinum-based doublet neoadjuvant therapy (n = 86, 86.9%), which included combination therapy with immunotherapy in four patients (4.0%). Targeted therapy was used in 17 patients (17.2%). The median length of neoadjuvant therapy was 1.97 months (IQR: 1.07–2.17). The median length of time from the cessation of neoadjuvant therapy to resection was 1.25 months (IQR: 0.81–1.97). The majority of resected tumors in this cohort were found to be composed of epithelioid histology (n = 75, 75.8%). These characteristics are summarized in **Table 1**. The peripheral blood marker characteristics are summarized in **Table 1**.

**Table 1:** Multivariable analysis of patients with epithelioid type malignant pleural mesothelioma reflecting characteristics significantly affecting overall survival.

Clinicopathologic Variable	Univariable		Multivariable	
	HR (95%CI)	*p value*	HR (95%CI)	*p value*
Age	1.06 (1.01-1.11)	0.028	1.02 (0.97-1.07)	0.500
				
Male Gender	0.38 (0.17-0.84)	0.017	0.61 (0.24-1.56)	0.301
Smoker	2.71 (1.38-5.32)	0.004	3.29 (1.41-7.66)	0.006
Asbestos Exposure	1.15 (0.57-2.31)	0.704		
FEV1 (pred)	0.99 (0.97-1.00	0.151	1.03 (0.95-1.12)	0.507
FVC (pred)	0.99 (0.97-1.00)	0.076	0.96 (0.89-1.04)	0.283
Zubrod				
0	Reference			
1	0.92 (0.48-1.78)	0.810		
2	2.16 (0.63-7.42)	0.223		
Clinical T status >2	1.33 (0.61-2.91)	0.479		
Clinical N status >0	0.86 (0.12-6.28)	0.878		
Pathologic T status >2	1.87 (0.89-3.95)	0.100	1.07 (0.44-2.62)	0.301
Pathologic N status >0	1.92 (0.99-3.72)	0.054	1.33 (0.52-3.39)	0.551
Surgical Procedure				
EPP	Reference			
PD	0.80 (0.42-1.52)	0.503		
SUVmax	1.01 (0.95-1.08)	0.700	1.03 (0.92-1.13)	0.733
Base Tumor Thickness (Sum)	1.00 (0.99-1.01)	0.735		
Base Max Tumor Area	0.99 (0.97-1.02)	0.656		
Change in Tumor Thickness	3.14 (1.62-6.09)	0.001	2.88 (1.31-6.30)	0.008
Lymphocyte count (pre-neoadjuvant therapy)	0.98 (0.64-1.50)	0.929		
Neutrophil count (pre-neoadjuvant therapy)	1.04 (0.94-1.15)	0.426		
Platelet count (pre-neoadjuvant therapy)	1.00 (1.00-1.00)	0.048	Collinearity	
PLR (pre-neoadjuvant therapy)	1.00 (1.00-1.00)	0.865		
NLR (pre-neoadjuvant therapy)	1.00 (0.95-1.05)	0.913		
PLR (post-neoadjuvant therapy)	2.85 (1.48-5.48)	0.002	1.49 (0.73-3.05)	0.276
NLR (post-neoadjuvant therapy)	1.89 (0.99-3.61)	0.055	1.51 (0.67-3.43)	0.322
Change in PLR	1.76 (0.93-3.33)	0.082	Collinearity	
Change in NLR	1.08 (0.57-2.04)	0.813		

HR, hazard ratio; EPP, extrapleural pneumonectomy; PD, pleurectomy and decortication; PLR, platelet to lymphocyte ratio; NLR, neutrophil to lymphocyte ratio.

**Figure 1:**
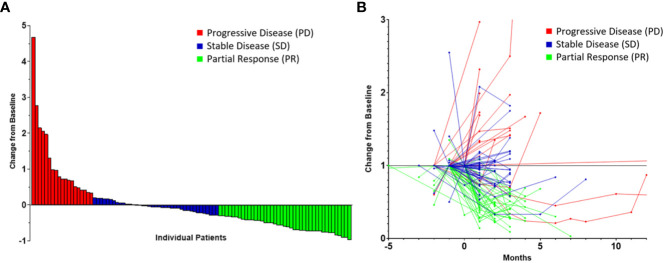
Radiomic data highlighting tumor response to neoadjuvant therapy in patients with malignant pleural mesothelioma stratified by response according to the modified RECIST criteria represented as a waterfall plot **(A)** and as a spaghetti plot **(B)**. Time of 0 month represents the initiation of neoadjuvant therapy.

The median pre-neoadjuvant therapy tumor thickness was 59.9 mm (IQR: 43.6–82.2), and the median thickness observed post-neoadjuvant therapy was 53.4 mm (IQR: 23.5–72.3). The median change in tumor thickness from the pre-neoadjuvant therapy CT scan to the most recent preoperative CT scan was -16.5% (IQR: -49.7 to 14.2). PR was observed in 41 patients (41.4%) while 39 patients (39.4%) had SD and 19 patients (19.2%) had PD (**Figures 1A, B**).

### 3.2 Survival analysis

**Figure 2:**
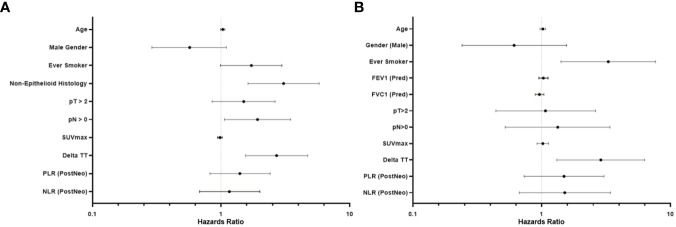
Forest plot representing the results of a multivariable analysis investigating survival beyond 20 months in patients with malignant pleural mesothelioma **(A)** and subgroup (epithelioid histology only) multivariable analysis **(B)**.

**Figure 3:**
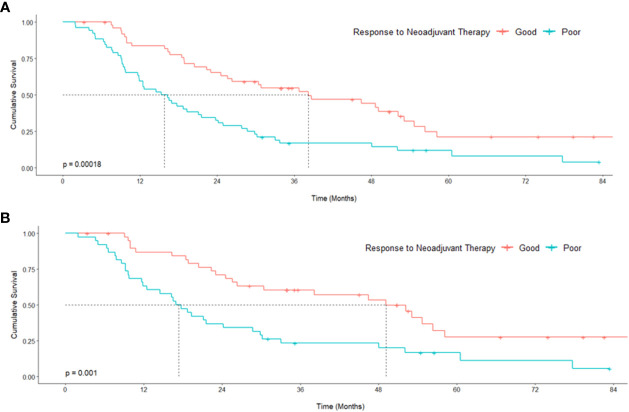
Kaplan–Meier curves representing survival analysis of a cohort of patients with malignant pleural mesothelioma **(A)** and subgroup [epithelioid histology only **(B)**] stratified around the median tumor response to neoadjuvant therapy. p values originate from a log-rank test comparing median survival.

Following univariate analyses, age, gender, smoking status, histology, pathologic nodal status greater than 0, and change in tumor thickness from neoadjuvant therapy met the criteria for inclusion in the multivariable Cox regression analysis. The regression analysis showed that patients with non-epithelioid MPM has a significant risk of mortality [hazard ratio (HR): 3.06, 95% confidence interval (CI): 1.62–5.78, p = 0.001]. Patients with pathologically proven nodal disease also were found to be at a survival disadvantage (HR: 1.92, CI: 1.06–3.48, p = 0.031). A change in tumor thickness that represented a response to neoadjuvant therapy inferior to the median response (tumors that decreased in size by 16.5% or less) was also associated with a greater risk of mortality (HR: 2.70, CI: 1.55–4.72, p < 0.001). The results of this analysis are summarized in **Table 2** and graphically represented in **Figure 2A**. Furthermore, Kaplan–Meier analysis showed that patients with a response inferior to a 16.5% decrease in thickness or disease progression on neoadjuvant therapy had a median survival of 15.77 months (CI: 11.83–23.78), while those with a response better than a 16.5% decrease in tumor thickness achieved a median survival of 38.17 months (CI: 25.59–54.66, p < 0.001, **Figure 3A**).

**Table 2:** Multivariable analysis of patients with malignant pleural mesothelioma reflecting characteristics significantly affecting overall survival.

Clinicopathologic Variable	Univariable		Multivariable	
	HR (95% CI)	p value	HR (95% CI)	p value
**Age**	1.07 (1.02-1.11)	0.003	1.03 (0.99-1.07)	0.354
**Male gender**	0.43 (0.22-0.84)	0.013	0.57 (0.29-1.10)	0.093
**Smoker**	2.50 (1.43-4.37)	0.001	1.72 (0.99-2.97)	0.054
**Asbestos exposure**	1.19 (0.64-2.18)	0.584		
**FEV1 (pred)**	1.00 (0.98-1.01)	0.81		
**FVC (pred)**	1.00 (0.98-1.01)	0.554		
Zubrod				
0	Reference			
1	1.04 (0.59-1.79)	0.916		
2	2.05 (0.61-6.91)	0.249		
3	2.73 (0.36-20.82)	0.332		
Histology				
Epithelioid	Reference		Reference	
Other (Sarcomatous/Biphasic)	2.01 (1.10-3.68)	0.023	3.06 (1.62-5.78)	0.001
**Clinical T status >2**	1.30 (0.71-2.38)	0.397		
**Clinical N status >0**	1.32 (0.41-4.22)	0.642		
**Pathologic T status >2**	1.82 (0.98-3.40)	0.06	1.50 (0.85-2.63)	0.162
**Pathologic N status >0**	1.44 (0.84-2.47)	0.182	1.92 (1.06-3.48)	0.031
Surgical procedure				
EPP	Reference			
PD	0.89 (0.52-1.52)	0.662		
**SUVmax**	1.03 (0.99-1.07)	0.172	0.98 (0.94-1.02)	0.354
**Base tumor thickness (Sum)**	1.00 (0.99-1.01)	0.897		
**Base max tumor area**	0.99 (0.97-1.01)	0.407		
**Change in tumor thickness**	3.33 (1.89-5.86)	<0.001	2.70 (1.55-4.72)	<0.001
**Lymphocyte count** **(pre-neoadjuvant therapy)**	0.92 (0.63-1.35)	0.686		
**Neutrophil count** **(pre-neoadjuvant therapy)**	1.01 (0.92-1.11)	0.786		
**Platelet count** **(pre-neoadjuvant therapy)**	1.00 (1.00-1.00)	0.082	Collinearity	
**PLR** **(pre-neoadjuvant therapy)**	1.00 (1.00-1.00)	0.908		
**NLR** **(pre-neoadjuvant therapy)**	0.99 (0.94-1.04)	0.677		
**PLR** **(post-neoadjuvant therapy)**	2.14 (1.32-3.47)	0.002	1.40 (0.82-2.41)	0.219
**NLR** **(post-neoadjuvant therapy)**	1.66 (1.03-2.66)	0.036	1.16 (0.68-2.00)	0.582
**Change in PLR**	1.54 (0.91-2.63)	0.111	Collinearity	
**Change in NLR**	1.35 (0.73-2.51)	0.339		

HR, hazard ratio; EPP, extrapleural pneumonectomy; PD, pleurectomy and decortication; PLR, platelet-to-lymphocyte ratio; NLR, neutrophil-to-lymphocyte ratio.

Univariate analyses of the subgroup of patients with epithelioid histology showed that age, gender, smoking status, pulmonary function test results, maximum standardized uptake value (SUVmax), change in tumor thickness, post-neoadjuvant therapy NLR and PLR met the inclusion criteria in our multivariable Cox regression analysis. The regression analysis revealed that smoking status had a significant effect on mortality (HR: 3.29, CI: 1.41–7.66, p = 0.006) as did change in tumor thickness from neoadjuvant therapy (HR: 2.88, CI: 1.31–6.30, p = 0.008, **Table 3**; **Figure 2B**). In this subgroup, Kaplan–Meier analysis showed that patients with a poor response to neoadjuvant therapy achieved a median survival of 17.33 months (CI: 11.86–29.83), while those with a good response attained a median survival of 49.11 months (CI: 26.31–Not Reached, p = 0.001, **Figure 3B**).

**Table 3:** Multivariable analysis of patients with epithelioid type malignant pleural mesothelioma reflecting characteristics significantly affecting overall survival.

Clinicopathologic Variable	Univariable		Multivariable	
	HR (95%CI)	*p value*	HR (95%CI)	*p value*
**Age**	1.06 (1.01-1.11)	0.028	1.02 (0.97-1.07)	0.500
**Male Gender**	0.38 (0.17-0.84)	0.017	0.61 (0.24-1.56)	0.301
**Smoker**	2.71 (1.38-5.32)	0.004	3.29 (1.41-7.66)	0.006
**Asbestos Exposure**	1.15 (0.57-2.31)	0.704		
**FEV1 (pred)**	0.99 (0.97-1.00	0.151	1.03 (0.95-1.12)	0.507
**FVC (pred)**	0.99 (0.97-1.00)	0.076	0.96 (0.89-1.04)	0.283
**Zubrod**				
0	Reference			
1	0.92 (0.48-1.78)	0.810		
2	2.16 (0.63-7.42)	0.223		
**Clinical T status >2**	1.33 (0.61-2.91)	0.479		
**Clinical N status >0**	0.86 (0.12-6.28)	0.878		
**Pathologic T status >2**	1.87 (0.89-3.95)	0.100	1.07 (0.44-2.62)	0.301
**Pathologic N status >0**	1.92 (0.99-3.72)	0.054	1.33 (0.52-3.39)	0.551
**Surgical Procedure**				
EPP	Reference			
PD	0.80 (0.42-1.52)	0.503		
**SUVmax**	1.01 (0.95-1.08)	0.700	1.03 (0.92-1.13)	0.733
**Base Tumor Thickness (Sum)**	1.00 (0.99-1.01)	0.735		
**Base Max Tumor Area**	0.99 (0.97-1.02)	0.656		
**Change in Tumor Thickness**	3.14 (1.62-6.09)	0.001	2.88 (1.31-6.30)	0.008
**Lymphocyte count** **(pre-neoadjuvant therapy)**	0.98 (0.64-1.50)	0.929		
**Neutrophil count** **(pre-neoadjuvant therapy)**	1.04 (0.94-1.15)	0.426		
**Platelet count** **(pre-neoadjuvant therapy)**	1.00 (1.00-1.00)	0.048	Collinearity	
**PLR** **(pre-neoadjuvant therapy)**	1.00 (1.00-1.00)	0.865		
**NLR** **(pre-neoadjuvant therapy)**	1.00 (0.95-1.05)	0.913		
**PLR** **(post-neoadjuvant therapy)**	2.85 (1.48-5.48)	0.002	1.49 (0.73-3.05)	0.276
**NLR** **(post-neoadjuvant therapy)**	1.89 (0.99-3.61)	0.055	1.51 (0.67-3.43)	0.322
**Change in PLR**	1.76 (0.93-3.33)	0.082	Collinearity	
**Change in NLR**	1.08 (0.57-2.04)	0.813		

HR, hazard ratio; EPP, extrapleural pneumonectomy; PD, pleurectomy and decortication; PLR, platelet to lymphocyte ratio; NLR, neutrophil to lymphocyte ratio.

## 4 Discussion

We report the survival probability of patients with MPM deemed to have undergone the “best” mesothelioma operation as judged by an operating surgeon, namely, maximum complete reduction, following neoadjuvant therapy. We found that patients with non-epithelioid MPM, nodal involvement, and less than 16.5% radiographic response to neoadjuvant therapy were at greater risk of early mortality following resection; the mortality of this group was similar to mesothelioma outcomes achieved without surgery. The median length of neoadjuvant chemotherapy in this group was 2 months, which permitted an investigation of pretherapy to presurgery tumor change, accounting for surgical decision-making. In the subgroup analysis of patients with epithelioid MPM, we found that smoking status and the lack of response to neoadjuvant therapy were associated with poor survival probability. In both our main analysis and our subgroup analysis, we observed that patients who underwent MCR following at least 16.5% radiographic response to neoadjuvant therapy obtained a survival benefit twice as long compared to those who did not respond to neoadjuvant therapy. This is a finding of significant clinical importance in such a rapidly lethal disease as MPM. Additionally, it provides a more powerful prediction of outcomes than nodal status and may more accurately represent cancer behavior and biology.

While patients with MPM can obtain meaningful outcomes from multimodal therapy, patient selection for resection continues to be intricately related to a compilation of factors. As we have observed, disease-specific factors should inform surgical decisions that mirror patient’s wishes, considering the significant convalescence associated with resection. Specifically in patients with epithelioid MPM, who are often referred for surgical consideration, who may have a median survival of 15.8 months following poor response to neoadjuvant therapy. Considering the significant survival benefit achieved with nivolumab plus ipilimumab (1) (median survival of 18.1 months) in patients with unresectable MPM, further investigations must consider the use of immunotherapy in patients who may have responded poorly to neoadjuvant chemotherapy and who may not benefit from surgical resection.

The current landscape in trimodality therapy, which includes resection, is rapidly evolving (12). Developments of neoadjuvant and maintenance protocols, with combination therapy or immunotherapy alone, are currently enrolling (13) or have recently completed accrual (14). Furthermore, neoadjuvant radiotherapy has also undergone investigation (15) with an ongoing trial exploring the benefits associated with oligofractionated radiotherapy followed by resection (15). Importantly, as meaningful survival benefits are obtained with systemic therapy in unresectable disease (16), the value of surgical resection is also being evaluated in clinical trials such as MARS2 comparing chemotherapy with neoadjuvant chemotherapy plus surgery (17). Advancements are also reported with novel systemic therapy such as T-cell immunoreceptor with immunoglobulin and ITIM domain (TIGIT) blockade (18).

This study is associated with limitations, which relate to the heterogeneity in surveillance imaging in this cohort; however, we were able to obtain tumor measurements from all patients included prior to the initiation of neoadjuvant therapy and prior to the resection. Similarly, we noted that using clinical stage was not reliable, considering the significant upstaging following resection, and thus, we decided to use pathological tumor and nodal statuses in order for our multivariate models to be adequately adjusted. Additionally, as represented in **Figure 1B**, there was a small group of patients who initially appeared to have a great response to systemic therapy who ultimately had progressive disease. This group may benefit from tailored therapy. Furthermore, the overall survival was calculated from the time of neoadjuvant therapy initiation rather than the date of diagnosis in order to alleviate additional heterogeneity in this cohort; however, this may not reflect true overall survival. Interobserver variability in measuring radiographic tumor change was minimized by having a single researcher performing the assessments, blinded to survival data at the time of the measurement. In conclusion, cytoreductive operations should be considered with increased carefulness in patients who do not respond to neoadjuvant therapy, as defined by radiographic metrics, which can serve as a possible surrogate for cancer biology. In the context of suboptimal response to neoadjuvant therapy, the patient’s wishes and quality of life must be considered prior to the pursuit of operative management. The involvement of patients in clinical trials or prospective treatment protocols must continue to be encouraged in order to further enhance therapeutic strategies in MPM.

## Data availability statement

The raw data supporting the conclusions of this article will be made available by the authors, without undue reservation.

## Ethics statement

The studies involving human participants were reviewed and approved by MDACC’s Institutional Review Board with a waiver of individual informed consent (PA 2019-1197). Written informed consent for participation was not required for this study in accordance with the national legislation and the institutional requirements.

## Author contributions

ND: conceptualization, investigation, visualization, methodology, writing–original draft, writing–review and editing. NZ and DM: visualization, methodology, writing–original draft, writing–review and editing. HF, JO, KT, EP, JZ, PL, MBA, and MA: investigation, writing–review and editing. WH, RM, RR, DR, JR, SS, and AV: patient accrual, writing–review and editing. AW: pathology review, writing–review and editing. AT: resources, funding acquisition, writing–review and editing. CH and BS: conceptualization, investigation, visualization, resources, supervision, funding acquisition, methodology, writing–original draft, project administration, writing–review and editing. All authors have contributed to the final article. All authors contributed to the article and approved the submitted version.
